# I RBH - First Brazilian Hypertension Registry

**DOI:** 10.5935/abc.20160120

**Published:** 2016-08

**Authors:** Paulo César Brandão Veiga Jardim, Weimar Kunz Sebba Barroso de Souza, Renato Delascio Lopes, Andréa Araújo Brandão, Marcus V. Bolívar Malachias, Marco Mota Gomes, Heitor Moreno Júnior, Eduardo Costa Duarte Barbosa, Rui Manoel dos Santos Póvoa

**Affiliations:** 1 Universidade Federal de Goiás, Goiânia, GO - Brazil; 2 Universidade Federal de São Paulo, São Paulo, SP - Brazil; 3 Universidade Estadual do Rio de Janeiro, Rio de Janeiro, RJ - Brazil; 4 Faculdade de Ciências Médicas de Minas Gerais, Belo Horizonte, MG - Brazil; 5 Universidade Estadual de Alagoas, Alto Cruzeiro Arapiraca, AL - Brazil; 6 Universidade Estadual de Campinas, Campinas, SP - Brazil; 7 Instituto de Cardiologia de Porto Alegre, Porto Alegre, RS - Brazil

**Keywords:** Evidence-Based Practice, Hypertension, Risk Factors, Multicenter Studies as Topic, Epidemiology, Prevalence

## Abstract

**Background:**

A registry assessing the care of hypertensive patients in daily clinical practice in public and private centers in various Brazilian regions has not been conducted to date. Such analysis is important to elucidate the effectiveness of this care.

**Objective:**

To document the current clinical practice for the treatment of hypertension with identification of the profile of requested tests, type of administered treatment, level of blood pressure (BP) control, and adherence to treatment.

**Methods:**

National, observational, prospective, and multicenter study that will include patients older than 18 years with hypertension for at least 4 weeks, following up in public and private centers and after signing a consent form. The study will exclude patients undergoing dialysis, hospitalized in the previous 30 days, with class III or IV heart failure, pregnant or nursing, with severe liver disease, stroke or acute myocardial infarction in the past 30 days, or with diseases with a survival prognosis < 1 year. Evaluations will be performed at baseline and after 1 year of follow-up. The parameters that will be evaluated include anthropometric data, lifestyle habits, BP levels, lipid profile, metabolic syndrome, and adherence to treatment. The primary outcomes will be hospitalization due to hypertensive crisis, cardiocirculatory events, and cardiovascular death, while secondary outcomes will be hospitalization for heart failure and requirement of dialysis. A subgroup analysis of 15% of the sample will include noninvasive central pressure evaluation at baseline and study end. The estimated sample size is 3,000 individuals for a prevalence of 5%, sample error of 2%, and 95% confidence interval.

**Results:**

The results will be presented after the final evaluation, which will occur at the end of a 1-year follow-up.

**Conclusion:**

The analysis of this registry will improve the knowledge and optimize the treatment of hypertension in Brazil, as a way of modifying the prognosis of cardiovascular disease in the country.

## Introduction

Hypertension is a polygenic disease of multifactorial etiology, characterized by sustained increased blood pressure (BP) levels.^1^ It is currently the main risk factor for cardiovascular diseases (CVDs), which are the most frequent causes of death in Brazil and worldwide.^1-3^ Hypertension is also associated with target-organ (kidney, heart, brain) damage and other comorbidities such as dyslipidemia and type 2 diabetes,^1^ which in turn also increase the risk of cardiovascular events.

A classic meta-analysis that evaluated more than 1 million individuals in 61 observational studies concluded that the mortality associated with CVDs increases progressively in a linear, continuous, and independent fashion with increases in BP levels above 115 / 75 mmHg.^4^

The INTERHEART study analyzed the risk factors for acute myocardial infarction (AMI) in 52 countries (six of which in Latin America), including 15,000 cases of patients with first AMI and a similar number of control individuals.^5,6^ The increased risk of AMI in Latin America was associated with hypertension (odds ratio [OR] 2.48; 95% confidence interval [95%CI], 2.03-3.04), diabetes (OR 2.45; 95%CI, 1.86-3.24), smoking (OR 2.35; 95% CI, 1.92-2.87), dyslipidemia - apoB/apoA1 (OR 2.79; 95% CI, 1.85-4.23), and abdominal obesity (OR 2.41; 95% CI, 1.79-3.25). Considering that these are all modifiable risk factors, the INTERHEART study alerted to the need for implementation of preventive strategies throughout Latin America.

The prevalence of hypertension in Brazil is similar to that in other countries. Surveys conducted in the Brazilian population point out to a prevalence of hypertension of approximately 30%.^7,8^ Considering BP values ≥ 140 / 90 mmHg, 22 studies found prevalences between 22.3% and 43.9% (mean 32.5%), with a higher prevalence in men compared with women, and in elderly individuals.^9^

A study in which 613 patients in the city of São Paulo were interviewed by phone found a 32% prevalence of hypertension among the respondents. Of these, 89% reported current treatment for hypertension but only 35% mentioned proper control of BP levels.^10^ Another study conducted in São José do Rio Preto in 1,717 individuals found a prevalence of hypertension of 25.2% and reported as associated factors the presence of advanced age, low educational level, and obesity.^11^ Among the treated hypertensive individuals, 52.4% showed adequate BP control, but only 34.3% of the overall treated and untreated hypertensive individuals had controlled BP levels.

Since hypertension is multifactorial, frequent in the adult population, and commonly associated with other cardiovascular risk factors and/or target-organ damage, it is an important public health problem. When the diagnosis of hypertension is established early, and the goals of BP control are met, the treatment of the disease may be effective; however, when left untreated, hypertension can cause severe complications and follow a slow and silent course.^1^

Data from the National Health and Nutrition Examination Survey (NHANES) collected between 2009 and 2012 have shown that 84.7% of the surveyed adults with hypertension were aware of the diagnosis of the disease and that the percentage of patients undergoing antihypertensive treatment was 76.5%, with a 54.1% rate of BP control. These numbers demonstrate a significant improvement in the diagnosis and control of hypertension in the American population.^12,13^

Lifestyle modifications lead to decreases in BP and cardiovascular mortality.^14-16^ Even though consistently recommended for prevention and treatment of hypertension, lifestyle modifications are difficult to implement from the patient's perspective and, often, also from the physician's perspective, who is frequently unable to rely on the support of a multiprofessional team.

With the objective of achieving proper BP control and consequently decreasing the deaths associated with CVD, it is essential to know the prevalence of hypertension in Brazil, clarify how the care and treatment of hypertensive patients are being conducted in daily clinical practice, and understand the role of multidisciplinary teams in this scenario.

## Objectives

### Main objective

- To document the current clinical practice for the treatment of hypertension in Brazil.

### Secondary objectives

- Stratify additional cardiovascular risks, considering associated comorbidities, target-organ damage, and presence of manifested cardiovascular disease;
- Analyze the profile of tests routinely requested for patients with hypertension;
- Verify the proportion of patients receiving antihypertensive treatment who are within the goals recommended by the Brazilian Society of Cardiology;
- Estimate the adherence to proposed pharmacological and non-pharmacological therapies using the Morisky scale;
- Evaluate the presence of a multiprofessional team in the regular care of the patients and, when present, the effectiveness of its performance in controlling the BP levels;
- Assess eventual differences in the approach to hypertensive patients in private and public centers.

## Methods

### Study design and population

This first Brazilian registry of hypertension will be a prospective, observational, multicenter, national study in which the subjects will be screened and evaluated in internal medicine and specialized outpatient clinics during a follow-up of 12 months.

The study will include individuals undergoing primary or secondary prevention, diagnosed with hypertension and meeting the inclusion criteria. After the subjects sign a free and informed consent form (FICF), their medical records will be analyzed, and they will be interviewed for completion of the electronic clinical charts (electronic case report form, eCRF) for inclusion in the study.

A follow-up visit will be scheduled between 6 months and 1 year from the inclusion in the study. In the event of more than one visit during this period, this additional visit may also be documented as an intermediate visit.

### Study population

Patients older than 18 years, with a diagnosis of hypertension for at least 4 weeks.

### Inclusion criteria

Signature of the FICF; age above 18 years; diagnosis of hypertension for at least 4 weeks, with a systolic BP (SBP) ≥ 140 mmHg and/or diastolic BP (DBP) ≥ 90 mmHg, measured with the participant seated and according to the VI Brazilian Hypertension Guidelines (*VI Diretrizes Brasileiras de Hipertensão*) or use of antihypertensive medication; and regular registration in the participating center/institution.

### Exclusion criteria

Renal failure requiring dialysis treatment; hospital admission at the moment of inclusion or in the previous 30 days; hemodynamic instability requiring vasoactive drugs in the previous 30 days; functional class III or IV heart failure; pregnancy and/or nursing; severe hepatic disease; psychiatric diseases preventing compliance with the protocol; history of stroke or AMI up to 30 days from the inclusion in the study; severe diseases according to the researcher's evaluation; neoplasms with a survival prognosis below 1 year.

### Criteria for interruption of the study

Individuals who wish to interrupt the participation in the study or who during follow-up present one or more exclusion criteria.

### Clinical evaluation

**The following clinical data will be assessed:**
- Family history of premature coronary artery disease (FH CAD, including a first-degree relative with a history of CAD before the age of 55 years if men or 65 years if women);
- Smoking, considering as smokers those individuals who routinely smoke one or more cigarettes or who routinely smoked up to 1 year before inclusion, and as previous smokers those individuals who stopped smoking more than 1 year before inclusion;
- Alcoholism, considering as current alcoholism the consumption of at least 30 g of ethanol for men (equal to approximately two cans of beer with 365 mL, two glasses of wine of 150 mL, or two doses of whiskey with 50 mL) and 15 g of ethanol for women (the equivalent of half of the doses described for men) at least three times a week, and as previous alcoholism the interruption of the habit for at least 1 year;
- Physical activity, considering as regularly performed those physical activities performed for at least 30 minutes three times a week;
- Menopause, characterized by the absence of menstrual periods for at least 1 year;
- Weight and height, measured with an anthropometric scale;
- Body mass index (BMI), calculated with the formula weight divided by the square of the height. Will be considered as eutrophic those individuals with a BMI < 25 kg/m^2^ and > 18 kg/m^2^, as overweight those with a BMI ≥ 25 kg/m^2^ and < 30 kg/m^2^, and as obese those with a BMI ≥ 30 kg/m^2^;
- Waist circumference (WC), measured with an inelastic measuring tape at the middle distance between the anterior superior iliac crest and the lower edge of the last costal arch with the patient standing and at the end of expiration;
- BP, measured with any type of regulated sphygmomanometer used routinely at the center, with two measures taken with the subject seated with an interval of at least 1 minute between each. Hypertension will be considered as the routine use of antihypertensive drugs or measurement of office BP equal to or above 140 x 90 mmHg. Individuals will be considered as having resistant hypertension when presenting uncontrolled BP levels despite the use of three classes of synergistic antihypertensive drugs in optimized doses, ideally including a diuretic; or if using four antihypertensive classes or more to achieve BP control;^17^
- Dyslipidemia, considered present in subjects using any hypolipidemic drug or with an LDL-c measurement above 160 mg/dL, and/or triglyceride level above 150 mg/dL, and/or HDL-c level below 40 mg/dL in men and 50 mg/dL in women, and/or total cholesterol above 200 mg/dL;
- Metabolic syndrome (MS), which will be diagnosed according to the International Diabetes Federation (IDF) criteria, detailed in Table 1, except for WC measurements, which will be considered ≥ 102 cm in men and ≥ 88 cm in women.

**Table 1:** IDF Diagnostic criteria for metabolic syndrome^18^

**Criteria**	**Definition**	
Waist circumference	Men	Women
White Europeans and Blacks	≥ 94 cm	≥ 80 cm
South Asians, Native Americans, and Chinese	≥ 90 cm	≥ 80 cm
Japanese	≥ 85 cm	≥ 90 cm
Triglycerides	≥ 150 mg/dL or treatment for hypertriglyceridemia	
HDL cholesterol	Men < 40 mg/dL	Women < 50 mg/dL
Blood pressure	Systolic ≥ 130 mmHg	Diastolic ≥ 85 mmHg
	or anti-hypertensive treatment	
Fasting blood glucose	≥ 100 mg/dL or DM treatment	

The diagnosis of metabolic syndrome includes the occurrence of abdominal obesity as an essential condition, and two or more of the remaining criteria. DM: diabetes.

### Clinical outcomes

#### Primary outcomes

The primary outcomes will include hospital admission for at least 24 hours due to hypertensive crisis; cardiovascular event (acute coronary syndrome [ACS] and/or stroke / transient ischemic attack [TIA]) documented in medical records or summary of hospital discharge; cardiovascular death (due to ACS, stroke / TIA, heart failure) confirmed by descriptions in the medical records and/or death certificate.

#### Secondary outcomes

The secondary outcomes will include hospitalization due to decompensated heart failure documented in medical records or summary of hospital discharge, and requirement of dialysis therapy.

### Recruitment

The patients will be recruited from outpatient clinics, and their participation in the study will begin after they sign the FICF previously approved by the Research Ethics Committee (*Comitê de Ética em Pesquisa*, CEP).

In the inclusion visit, the following information will be collected: demographic data, personal history, family history, hypertension history, BP assessment, medications in use within the previous 30 days, laboratory data and electrocardiogram (ECG) in the last 6 months (if available), analysis of other relevant tests in the investigator's opinion and performed in the previous year, lifestyle questionnaire, ambulatory BP in the previous 6 months (if available), and treatment adherence assessed with the Morisky scale.

In the follow-up visit, the evaluating data will include BP measurements, medications in use, estimate of adherence to pharmacological and non-pharmacological treatment using the Morisky scale, hospitalization due to CVD (hypertensive crisis, AMI, angina, stroke, TIA, heart failure), beginning of dialysis, and death.

### Subgroup analysis

A protocol subgroup including 15% of the sample will undergo a noninvasive assessment of the central BP with a validated Mobil-O-Graph equipment (DINA MAP CARDIOS - I.E.M. GmbH, Stolberg, Germany) in the inclusion and follow-up visits.

The central hemodynamic parameters will be obtained using a noninvasive method that verifies simultaneously the pulse wave of the arterial blood flow, allowing analysis of the BP, central systolic pressure (cSP), pulse wave velocity (PWV), and augmentation index (AIx) by the method ARCsolver, Austrian Institute of Technology. This method determines the cSP based on brachial pulse waves recorded with a cuff connected to the Mobil-O-Graph, the oscillometric device that records the BP. The records will be obtained by inflating the cuff for 10 seconds at the DBP level, using conventional cuffs for adults (available in the sizes 24-34 cm and 32-42 cm) and a high-fidelity MPX50550 pressor sensor (Freescale Inc., Tempe, AZ, USA). The method considers the influence of arterial impedance using a transfer function, as well as the hemodynamics of the aorta using a mathematic method. The Mobil-O-Graph is a device that allows simultaneous measurement of central and peripheral pressure. Depending on the protocol adopted, these measurements may be carried out at pre-established intervals. The same preparation methodology described for the measurement of casual BP will be adopted, with the use of a suitable cuff, according to the circumference of the chosen arm.

Central parameters are usually performed in a single evaluation. With this equipment, the assessments can be performed by several protocols with multiple measurements.

For those research centers with patients participating in this subgroup, the following variables will be collected and entered in the registry's eCRF: cSP, central diastolic pressure (cDP), PWV, AIx (corrected for 75% of the heart rate), and peripheral arterial resistance.

This analysis aims to offer an understanding of the reality of the Brazilian population regarding the information mentioned above, and to correlate different classes of antihypertensive drugs in terms of action on peripheral BP levels and on variables regulating the central BP control.

### Statistical analysis and sample size

A sample of 3,000 individuals will be sufficient to estimate prevalences of 5% (or greater) with an absolute sample error (precision) of 2% (or greater) and a 95%CI considering a 20% loss to follow-up.^19^ Survival curves will be calculated with the product-limit Kaplan-Meier method^20^ and compared with the log-rank test proposed by Mantel,^21^ followed by Sidak's multiple comparisons test, when appropriate. Survival probabilities will be estimated with 95%CI using log-log transformation.^22^ Cox univariate and multivariate analyses (controlling for factors of interest such as comorbidities, sociodemographic variables, demographic regions, age, etc.) will be conducted to identify factors associated with the survival outcomes evaluated. The proportional hazards assumptions and functional form (for continuous covariables) in Cox regression models will be assessed according to the Kolmogorov-type supremum test based on a sample of 1,000 simulations of residuals.^23^ If the linear form is rejected for a continuous covariate, the covariate will be categorized according to the cutoff points of clinical importance or according to distribution percentiles. Adjusted and unadjusted hazard ratios with corresponding 95%CIs will be estimated by Cox univariate and multivariate regression analyses, respectively. In addition, statistical analyses will be conducted in previously defined subgroups in different Brazilian regions (North, Northeast, Midwest, South, and Southeast) and according to the type of service performed (academic or non-academic, public or private).

If a violation of the assumption is observed, weighted Cox regression will be used.^24^ Before determining the multivariate model, a diagnosis of multicollinearity will be conducted according to the variance inflation factor (VIF). The cases confirmed as loss to follow-up will be censored at the date of the last follow-up / contact. Associations between categorical variables will be evaluated in 2x2 and greater than 2x2 contingency tables with Fisher's exact test and its extension, the Fisher-Freeman-Halton test, respectively. Unpaired Student's *t* test and analysis of variance (ANOVA) will be used to compare averages among groups of two or more than two, respectively. In cases of considerable data asymmetry, nonparametric alternatives will be applied: Wilcoxon's rank sum and Kruskal-Wallis tests, respectively.^25^ The normality of the data will be evaluated with a visual inspection of the histograms and/or application of the D'Agostino and Pearson's comprehensive normality test, when appropriate.^26^ Continuous variables will be described as mean ± standard deviation or median (interquartile range), when appropriate. Categorical variables will be described as numbers (percentages). In addition, statistical analyses will be conducted in previously defined subgroups. All probabilities of significance (p values) presented will be bilateral and values below 0.05 will be considered statistically significant. The data will be statistically analyzed with SAS 9.3 (Statistical Analysis System, Cary, NC, USA).

### Legal and ethical aspects

This registry is conducted in accordance with national and international resolutions such as the Declaration of Helsinki, Resolution CNS196/96 and all the complementary CNS/MS, ICH Good Clinical Practices guide(1996), and the Document of the Americas (2005).

Each clinical research center will submit this protocol, the FICFs, and all other applicable documents to the institutional CEP for analysis and approval prior to any procedure included in the registry.

### Organization of the study

#### Coordinating Center

The Brazilian Clinical Research Institute (*Instituto Brasileiro de Pesquisa Clínica*, BCRI) in São Paulo, Brazil, was responsible for the development of the protocol and operational coordination of the study, development and implementation of the electronic platform for data collection (electronic case report form), distribution of required materials to the research centers, preparation of regulatory dossier, newsletter, quality assurance of the collected data and organization of meetings for researchers, preparation of a monitoring plan, determination of training for the research centers, periodic monitoring visits, and remote study closure. The Institute for Teaching and Research of *Hospital do Coração* (*Instituto de Ensino e Pesquisa do Hospital do Coração*) will be responsible for maintaining the database and analyzing the data.

### Steering Committee

The Steering Committee will be responsible for the design, implementation, analysis of the study data and allocation of appropriate responsibilities to other committees of the study.

#### Research Centers/National Researchers

Andréa Araújo Brandão, Adriana de S. Thiago Papinutto, Álvaro Rabelo Jr., Antonio Felipe Sanjuliani, Antônio Carlos Sobral Sousa, Celso Amodeo, Cristiano Pederneiras Jaeger, Dalton Bertolim Précoma, Estêvão Lanna Figueiredo, Epotamenides Maria Good God, Evandro José Cesarino, Fábio Argenta, Fernando Augusto Alves da Costa, Gilberto Campos Guimarães Filho, Gustavo Freitas Feitosa, Helder José Antônio Miranda Abrantes, Iran Castro, José Lima Reis, Joaquim David Carneiro Neto, João Carlos Ferreira Braga, João Miguel Malta Dantas, João Soares Felício, João Roberto Gemelli, Jose Albuquerque de Figueiredo Neto, José Carlos Ayub, José Fernando Vilela Martin, Leila Beltrami Moreira, Lívia Figueira Avezum Oliveira, Luiz Bortolotto, Luciana Reis Katz Weiand, Luciano Marcelo Backes, Luiz Carlos Bodanese, Luiz César Nazário Scala, Márcio Kalil, Marco Mota Gomes, Margaret Assad Cavalcante, Maria das Dores de Oliveira Jehá, Mario Fritsch Neves, Pedro Barros, Renato Delascio Lopes, Roberto Dischinger Mirand, Rodrigo Pinto Pedrosa, Rodrigo Ricardo Silva da Costa, Rogério Toshiro Passos Okawa, Rui Manoel dos Santos Póvoa, Sergio Kaiser, Thiago de Souza Veiga Jardim, Wladmir Saporito, Weimar Sebba Barroso, Wilson Nadruz Junior.

## Conclusion

A better understanding of the way that hypertensive patients are treated in Brazil will allow the implementation of policies aimed at improving the treatment of hypertension, contributing to changes in the epidemiological profile and decrease in cardiovascular morbidity and mortality in the country.
